# The value of MRI in management of endometrial hyperplasia with atypia

**DOI:** 10.1186/s12957-020-1811-5

**Published:** 2020-02-10

**Authors:** Purushothaman Natarajan, Angela Vinturache, Richard Hutson, David Nugent, Timothy Broadhead

**Affiliations:** 1 Department of Obstetrics & Gynaecology, Liverpool Women’s Hospital, Liverpool, UK; 2 Department of Obstetrics & Gynaecology, St George’s Hospital, St George’s University Hospitals NHS Foundation Trust, London, UK; 3 Department of Gynaecologic Oncology, Leeds Teaching Hospitals, Leeds, UK

**Keywords:** Endometrial hyperplasia, Myometrial invasion, Endometrial cancer, Magnetic resonance imaging, Ultrasonography, Sensitivity and specificity

## Abstract

**Background:**

The value of the magnetic resonance imaging (MRI) in the assessment of women with endometrial hyperplasia and its role in diagnosis of myometrial invasion or coexistence of cancer is not known. This study aimed to evaluate the accuracy and usefulness of MRI in the management of patients diagnosed on endometrial biopsy with complex endometrial hyperplasia with atypia (CEHA).

**Methods:**

A retrospective study of 86 cases diagnosed with endometrial hyperplasia with atypia on the initial endometrial biopsy in a tertiary university teaching hospital between 2010 and 2015 was carried out. The MRI accuracy in predicting malignant changes and influence the clinical management was compared among women who had either pelvic MRI, transvaginal ultrasound (TVUS), or no additional imagistic studies.

**Results:**

MRI was performed in 24 (28%) and TVUS in 11 (13%)cases, while 51 (59%) women had no additional imagistic studies. In the group of women with no imaging studies, 26/51 (51%) were surgically treated and 8/26 (31%) were diagnosed with endometrial cancer (EEC) stage 1a. In the group of women who had TVUS, 5/11 (45%) were surgically treated and none was diagnosed with EEC. In the group of women who underwent an MRI examination, 20/24 (83%) were surgically treated. Among these, 11/20 (55%) were diagnosed with EEC, 7 had EEC stage 1a, and 4 had EEC stage 1b. Although MRI was able to identify malignant changes with a good sensitivity (91.7%), it had a low specificity in characterisation of malignant transformation (8%). MRI correctly identified 31% of the stage 1a and 33% of the stage 1b endometrial cancer.

**Conclusion:**

In this study, we found a potential diagnostic value of MRI for identifying malignant transformation in patients with CEHA. However, pelvic MRI has a rather weak predictive value of myometrial invasion in women with CEHA and concurrent EEC. The diagnostic and therapeutic benefits of MRI assessment in patients with CEHA need further validation.

## Introduction

Worldwide, there is an increase in the incidence of endometrial pathology that parallels the progressive ageing of the population and increase in the prevalence of obesity [1]. The spectrum of endometrial changes varies by architectural complexity and nuclear cytology. Among these, endometrial hyperplasia is defined as irregular proliferation of the endometrial glands with an increase in the gland to stroma ratio when compared with proliferative endometrium. Endometrial hyperplasia includes non-neoplastic entities (simple and complex hyperplasia without atypia) and precancerous intraepithelial neoplasms (complex endometrial hyperplasia with atypia, CEHA). Strong evidence demonstrates that endometrial hyperplasia is the precursor of endometrial cancer, and if left untreated, it can progress to cancer or may coexist with cancer [2–5]. Endometrial hyperplasia with atypia is the least common type of hyperplasia but is the type most likely to progress to type 1 endometrial carcinoma (EEC) (30–50%) [6–8], whereas simple hyperplasia without atypia is unlikely to progress to malignancy and progestogen therapy is usually recommended [9]. Not surprisingly, most women with CEHA undergo hysterectomy as primary treatment, but non-surgical management can also be effective [10].

Endometrial cancer is the most common gynaecological malignancy in the Western world and the fourth most common cancer among women [2, 11]. Despite the fact that endometrial carcinoma is the most common gynaecologic cancer, less is known about the incidence of its precursor lesion, endometrial hyperplasia. It is estimated, however, that the incidence of endometrial hyperplasia is at least three times higher than endometrial cancer. Current estimates report incidence of endometrial hyperplasia to be around 133–208 per 100,000 woman-years in Western countries [3] and 37/100,000 woman-years in Korea [12]. The incidence rates of the endometrial hyperplasia subtypes are 121 per 100,000 woman-years for non-atypical hyperplasia and 16.8 per 100,000 woman-years for atypical hyperplasia [3, 13].

Abnormal uterine bleeding is the most common presenting symptom of endometrial abnormalities, hyperplasia, or cancer. Investigation into the cause of bleeding and evaluation of abnormalities of the endometrial cavity poses a significant diagnostic challenge for radiologists and gynaecologists. The techniques commonly used to assess the endometrium in symptomatic women are transvaginal sonography (TVS) and endometrial biopsy [9], with equal sensitivities for detection of endometrial changes suggestive of endometrial carcinoma [14]. Few studies have been done to assess the merits of screening for detection of endometrial cancer in asymptomatic women [15]. Improvements in imaging technology over time have led to its increasingly widespread use in health care.

Computerised tomography (CT) and diffusion-weighted magnetic resonance imaging (MRI) may aid in the diagnosis of hyperplasia, although their role is not yet clear and, as such, they are not commonly used. However, evidence suggests that modern imaging may provide important tools in the accurate pre-treatment assessment of more advanced endometrial changes and may optimise treatment planning [16]. Studies have shown that CT scan can change management in only 4.3% of cases, thus rarely alters the management in patients with uterine neoplasm [17]. MRI appears to be of low value in predicting extra-uterine disease among uterine cancer patients with low grade disease, however, may help in identifying myometrial invasion and accurate cervical involvement that cannot be predicted clinically [9, 15, 17]. To date, there is little consensus on the use of MRI imaging in the routine preoperative assessment of endometrial malignancy, and practice varies largely among gynaecologists. Given the questionable utility of MRI in this disease, additional studies are required to define the use of this imaging test and its utility in pre-therapeutic assessment of endometrial lesions.

To this end, this study was designed to evaluate the role of pelvis MRI in the management and outcomes of complex endometrial hyperplasia with atypia, CEHA. The aim of the study was to find out whether MRI would alter the management of CEHA.

## Materials and methods

### Patient population

This retrospective study was drawn from the West Yorkshire and Humber NHS Deanery regional audit that assessed the use of MRI in CEHA. The electronic health records database of the St. James Hospital, Leeds Teaching Hospitals NHS Trust, UK, was searched to identify adult women who had a histologic diagnosis of endometrial hyperplasia with atypia between January 2010 and December 2015.

The database search identified 86 women with an initial histology diagnosis of atypical endometrial hyperplasia (focal or complex, CEHA) on the endometrial curettings performed within the time frame of the study. The diagnosis of atypical hyperplasia was based upon the presence of the microscopic features described in Additional file 1: Table S1. Clinical information and management decisions of these women were further abstracted from the electronic records and manual search of hand-held clinical notes. All patients were premenopausal or early postmenopausal.

### Methodology

**Fig. 1:**
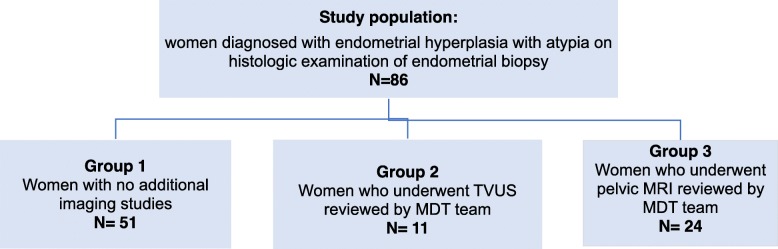
Flowchart of the study population

As part of clinical assessment, following initial presentation, all women included in the present study had pelvic US scans and endometrial biopsies [9], with endometrial hyperplasia with atypia diagnosed by histology examination of the biopsy product. In the endometrial surveillance follow-up, of the 86 cases, 51 had no further imaging studies (group 1), 11 women had pelvic US (group 2), and 24 women had a pelvic MRI (group 3). For women who had no additional imaging, the management was proposed and carried out based on the initial histology diagnosis and initial US scan of the pelvis. For the women who had follow-up pelvic US scans, the scan images and clinical details were reviewed by the Hysteroscopy Multidisciplinary Team (MDT), who made the management recommendations. Gynaecologic Oncology MDT made the management decisions for the women who had additional MRI scans, after review of scan images, clinical, and histological picture. The decision of additional imagistic studies was based on clinical assessment of the balance between benefit and potential harm. The flowchart of the study population is provided in Fig. 1.

Main outcome measure of this study was the correlation between the diagnosis of myometrial invasion suggested by pre-intervention MRI study and the subsequent histopathological findings following examination of the biopsy (curetting) or hysterectomy specimen. We compared MRI findings suggestive of myometrial invasion with the histology reports of surgical and/or biopsy specimens examined.

Secondary endpoint was to assess how additional pelvic assessment with TVUS or no further imagistic changed the management of women diagnosed with endometrial hyperplasia with atypia at the endometrial assessment at the presentation (initial endometrial histology).

### Ethical considerations

This study was compliant with the Health Insurance Portability and Accountability Act [18] and approved by the Institutional Audit Review Board of the Leeds Teaching Hospitals NHS Trust, with waiver of written informed consent.

To assess our results and their significance, we searched previous literature using PubMed, Embase, and Cochrane database of systematic reviews and reviewed publications to date on the topic.

### Statistical analysis

Descriptive statistics were produced for all three groups. Categorical data were presented as frequencies and percentage. Sensitivity, specificity, PPV, NPV to diagnose/predict malignant transformation for pelvis MRI, TVUS, and no imaging were calculated. Significance was accepted at *p* < 0.05 and all tests were two-sided. SPSS version 23.0 (IBM) was used for statistical computations.

## Results

Eighty-six women were included in the present study, with an average age of 60.4 ± 14.5 years (range 33–93 years old) and BMI of 43.5 ± 11.0 kg/m^2^ (range 26.1–56.0).

### Group 1: Women with no additional imaging studies

From the group of women who had no additional imaging (*n* = 51), 37 women had focal atypical changes of the endometrium and 14 women had a histology of complex atypical hyperplasia (CEHA) on the initial endometrial biopsy. The management decisions for these women were based on the pre-biopsy scans and histology results of the endometrial biopsies. Twenty-eight (55%) women from this group had medical management, 20 (39%) had surgical intervention, and 3 (6%) did not have any interventions. Medical management consisted in administration of progestogens, local intrauterine LNG-IUS (Mirena) and/or continuous oral, or injectable progesterone (medroxiprogesterone). Among women who had surgical intervention, 16 had total abdominal hysterectomy and bilateral salpingo-oophorectomy, one had vaginal hysterectomy, and one had total laparoscopic hysterectomy with bilateral salpingectomy. Women who had no further interventions had only focal histological changes of atypia.

For the 20 women who had surgical treatment, histology of the resected uteri showed no further evidence of hyperplasia or malignancy for 12 cases (60%), stage 1a grade 1 endometrial cancer (EEC) in 5 cases (25%), one case of simple hyperplasia (5%), one case of focal complex hyperplasia (5%), and one case of CEHA (5%). A summary of the histologic findings and interventions for these women is provided in the flowchart in Additional file 2: Figure S1.

Among the 14 women with an initial diagnosis of CEHA who did not undergo additional imaging, 6 had surgical intervention and 8 had medical management. The histology of surgical specimens in women with CEHA who underwent surgical management showed advanced changes to stage 1a grade 1 EEC for 3 women, and 1 woman had focal atypical complex hyperplasia, whereas two women persistent CEHA changes. In women who received medical management, follow-up biopsies showed progesterone effect on follow-up histology in 4 women and simple hyperplasia without atypia in 2 women, whereas 2 women did not have further biopsies at the time of the study.

**Fig. 2:**
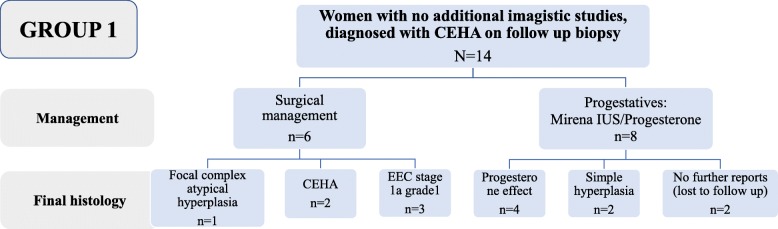
Diagnosis and management of women from group 1, with an initial diagnosis of CEHA, who had no additional imagistic studies

The management and histology findings in women with CEHA who had no additional imaging studies are summarised in Fig. 2.

Overall, a diagnosis of EEC was made in 5 women with atypical hyperplasia (10%, 5/51), among which 3 women (6%, 3/51) had an initial diagnosis of CEHA. In other words, in women with CEHA who had no imaging studies, an EEC was diagnosed in 21% of cases (3/14).

### Group 2: Women with pelvic US scans

**Fig. 3:**
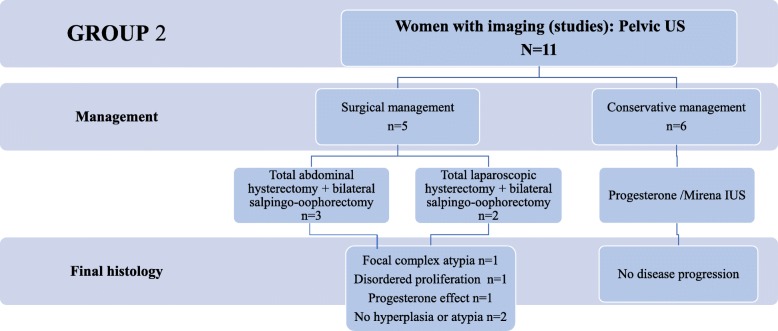
Diagnosis and management of women from group 2, who underwent additional pelvic TVUS scans

For the 11 women who had further assessment by pelvic TVUS scans, the scan images were evaluated by the MDT team of local consultants that included radiologist with expertise in gynaecological scanning, pathologist, and gynaecologists. No additional imaging studies were deemed necessary to aid the management for these women. From this group, 5 (45%, 5/11) women underwent surgical management and 6 (54%, 6/11) women had conservative management with progestatives or Mirena. Three of the women who had surgical management underwent total abdominal hysterectomy and bilateral salpingo-oophorectomy, whereas the other two had laparoscopic hysterectomy and bilateral salpingo-oophorectomy. The final histology for the women managed surgically showed focal complex atypia in one woman and benign changes in all the other cases. None of the women managed conservatively had disease progression (EEC) (0%, 0/11) on the subsequent histology examinations during the endometrial surveillance protocol. Figure 3 summarises the findings in this group of women.

### Group 3: Women with pelvic MRI studies

**Fig. 4:**
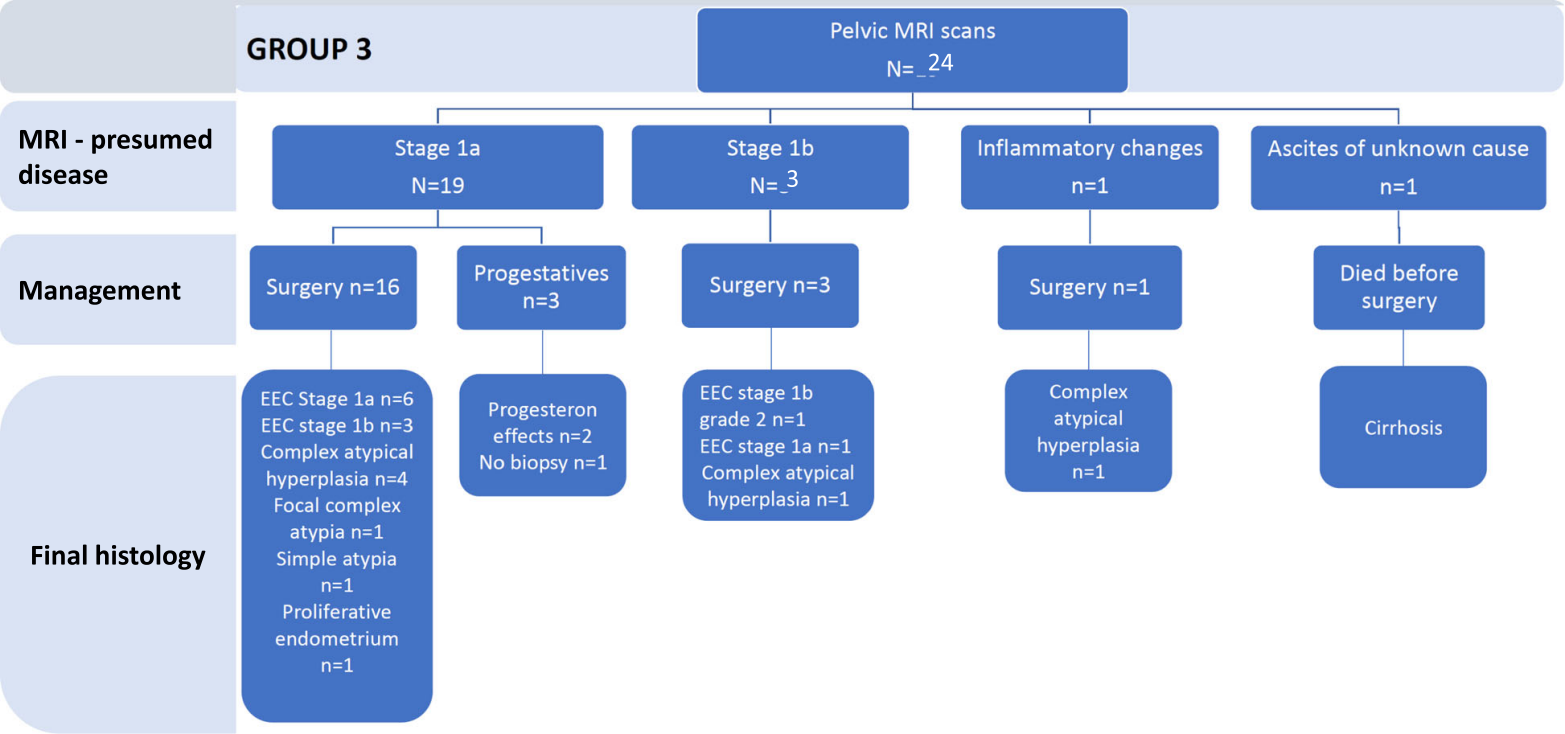
Diagnosis and management of women from group 3, who underwent additional pelvic MRI scans

MRI scan was proposed for further evaluation of myometrial invasion and extra-uterine disease for 24 women initially diagnosed with CEHA [19]. As shown in Fig. 4, based on the analysis of the MRI images, 19 women from this group were presumed to have EEC stage 1a (79%, 19/24), 3 were presumed EEC stage 1b disease (12.5%, 3/24), one case was predicted to have pelvic inflammatory disease (4%, 1/24), and one was diagnosed as ascites of unknown cause, requiring further investigations (4%, 1/24).

Among the 19 cases of presumed stage 1a disease on the pelvic MRI, 16 underwent surgical intervention and 3 medical management. Of the 16 women who had surgical management, final histology diagnosed stage 1a EEC in 6 cases, stage 1b EEC in 3 cases, CEHA in 4 cases, focal complex atypia, simple hyperplasia, and proliferative endometrium in one case, respectively. Of the 3 women who had medical management, 2 showed progesterone effect on subsequent biopsies, whereas 1 woman did not have any follow-up biopsy.

Of the three cases presumed to have EEC stage 1b disease on the MRI, one case was confirmed with stage 1b EEC and one case was diagnosed with stage 1a focal EEC, whereas for the third woman, histology examination showed complex atypical hyperplasia. The follow-up histology evaluation of the woman with inflammatory finding on the MRI found complex atypical hyperplasia. The woman in whom MRI identified ascites was diagnosed with hepatic cirrhosis. This patient died before the date of the surgery.

Overall, in this group, MRI suspected EEC in 21 cases (87%, 21/24), which was confirmed in 11 cases (46%, 11/24). Twenty women from this group underwent surgical treatment (83%, 20/24). A summary of MRI diagnoses, management, and follow-up histology in this group of women is shown in Fig. 4.

Among 86 women included in the study, 57% (49/86) had an initial diagnosis of CEHA. From the 16 women diagnosed with EEC (18%, 16/86), 14 (16%, 14/86) had an initial diagnosis of CEHA. Among 22 women who showed myometrial invasion of the MRI images, 11 were confirmed by pathology with EEC stage 1, either 1a or 1b disease, rendering a high sensitivity, of 100% (95% CI, 71.5–100.0) but low specificity, of only 15.4% (95% CI, 1.9–45.5) of MRI in predicting endometrial/myometrial invasion in women with an initial diagnosis of CEHA. The positive predictive value of MRI was 50.0% (95% CI, 44.2–55.7), the negative predictive values were 100.0%, and the accuracy was 54.1% (95% CI, 32.8–74.4).

## Discussion

This study assessed the performance of MRI in endometrial surveillance and accuracy in prediction of malignancy and uterine invasion in a population of perimenopausal women who had a diagnosis of endometrial hyperplasia with atypia at their evaluation of initial presentation for abnormal uterine bleeding. We calculated sensitivity and specificity of pre-intervention MRI in predicting the degree of endometrial anomaly and myometrial invasion.

This study shows that pelvic MRI has a potential diagnostic value for identifying a concurrent malignancy or malignant transformation in patients with CEHA. Although it correctly identified all cases of EEC among the CEHA patients, it had a false positive rate of 46%. In other words, we found that, although MRI was able to identify malignant changes with a good sensitivity, this investigation has a low specificity in characterisation of malignant transformation, misclassifying in excess almost half of the endometrial lesions. However, MRI appears less suited to potentially provide preoperative imaging biomarkers in early stages of malignant transformation and offer relevant information for risk stratification and individualised treatment and prognosis, as MRI correctly could identify only 58% of the stage 1a and 20% of the stage 1b endometrial cancer in the present study.

Notably, a higher proportion of patients in the MRI group were surgically treated. MRI examinations led to an increase by 33% in surgical interventions for CEHA. Nevertheless, the MRI group also had the highest proportion of final diagnosis of ECC among the surgically treated (55% versus 31% and 0% in the other patient groups). It may thus be argued that, although the percentage surgically treated were higher in the MRI group, the percentage of surgically treated patients who were likely to profit from the surgical treatment due to final ECC diagnosis was higher in the MRI group. As such, the potential diagnostic value of MRI for identifying patients in need of surgery is highly illustrated by these findings, thus increasing the value of MRI for patients with CEHA.

Several studies have been conducted over the past years to assess the predictive value of MRI in diagnosis of myometrial invasion or absence thereof in women with endometrial cancer with various results. Most studies have shown that preoperative pelvic MRI is a method with moderate sensitivity and specificity in identifying invasion to the myometrium in endometrial cancer and a rather weak predictive value when used to assess absence of myometrial invasion [20–24]. However, addition of MRI to preoperative assessment may lead to improved preoperative assessment, triage, and treatment of women with endometrial cancer [20, 22]. On the other hand, there is limited evidence on the predictive value of MRI in women with advanced endometrial hyperplasia. Thus, MRI in the diagnosis of hyperplasia is not commonly used.

Similar with our findings, a recent study from Ofinran and Balega [25] found that in women with an initial histologic diagnosis of CEHA, MRI had a better predictive value for invasion and performed poorly in predicting no invasion. Another study aiming to determine the utility and cost effectiveness of preoperative computed tomography (CT) in detecting the extent of disease in patients with high risk endometrial histology reported that a preoperative CT scan of women who have atypical endometrial hyperplasia or grade 1 endometrial cancer could alter management in 4.3% of cases [17]. However, there are no studies evaluating CT use for following up of women with endometrial hyperplasia when treated conservatively.

On the other hand, TVUS scan was able to correctly identify absence of malignant changes in the endometrium, with a sensitivity of 100%. In our study, pelvic scans showed no features suggestive of malignant transformation in any women in the group, which was confirmed by histology. The exact accuracy of TVUS in diagnose of endometrial hyperplasia is not known, and the reported sensitivity varies largely between 59.7 and 100% [26, 27]. Many studies generally define an endometrial thickness lower than 5.0 mm as the normal cut-off value in postmenopausal women, as endometrial thickness of 3.0–4.0 mm in postmenopausal women reduces the chance of endometrial cancer to less than 1% [28–30]. Whereas most studies relayed on endometrial thickness and its cut-off value, recent studies have shown that endometrial stripe abnormality may be a better predictor of endometrial hyperplasia in healthy premenopausal and perimenopausal women with and without abnormal uterine bleeding than simple measurement of endometrial thickness [31]. Transvaginal ultrasound may have a role in diagnosing endometrial hyperplasia in both pre- and postmenopausal women. Direct visualisation and biopsy of the uterine cavity using hysteroscopy are suggested when hyperplasia has been diagnosed in a polyp or in a hidden focal area [9]. Of note, there is no systematic correlation between the hysteroscopic features and the diagnosis of endometrial hyperplasia. Although recent studies report that hysteroscopy may have high sensitivity and negative predictive value in diagnosis of endometrial hyperplasia, there is still no consensus in the objective criteria for diagnosis nor it is known its value in follow-up of CEHA patients.

In this study, we used the data from three different cohorts of women diagnosed with atypical hyperplasia, who were randomised by the attending clinicians to further investigation and management based on the degree of endometrial abnormality. We have noted that in cases where atypia was focal or localised to a polyp, surgical intervention without any further investigations is feasible without increase in adverse outcomes. Undertaking further TVUS scans with review by the multidisciplinary team is a feasible alternative, that allows case selection more effectively, rather than subjecting all women diagnosed with CEHA to have MRI scans.

In view of the significant risk of an underlying or developing malignancy in women with CEHA, the usually recommended management is total abdominal hysterectomy, BSO, and washings [19]. There is limited evidence on the likelihood of long-term endometrial hyperplasia response to progestin therapy, especially for atypical hyperplasia. Recent data suggest that, in short term, most women with hyperplasia respond to progestogenic therapy and are not at increased risk of developing cancer. The patients with an unfavourable response to and a significant elevation in cancer risk can be identified on the basis of cytologic atypia [8]. Although endometrial carcinoma is undoubtedly the most important outcome, the rates of hysterectomy in our study were considerable and thus may have significant individual, societal, and economic impact. The overall rate of surgical intervention was 53% (45/86), with the highest rate in women who had endometrial surveillance by MRI, 84% (20/24). Others estimate that hysterectomy is performed in 75–80% of women with atypical hyperplasia [3]. Progestin therapy decreased the risk of hysterectomy in women with complex and atypical hyperplasia, respectively. It appears that the decision whether to follow with imagistic studies and attempt hormonal therapy with progestins or to proceed to hysterectomy is influenced by the perceived risk of progression to invasive carcinoma that each histology-based diagnosis carried. Our work would suggest that among women with a diagnosis of focal atypical hyperplasia a trial of progestin with strict surveillance for recurrence is relatively safe regarding risk of endometrial carcinoma. However, this strategy does not completely negate endometrial carcinoma risk. Whether women with endometrial hyperplasia would require ongoing progestin therapy for several years is still unknown.

### Strengths and limitations

Our study is one of the few that investigated the role of MRI in CEHA surveillance, assessing the accuracy of MRI in detection of endometrial cancer lesions associated or co-existent with hyperplasia. Although this is a small retrospective study, the clinical management decisions and recommendations were done in agreement with the contemporary guideline and best evidence clinical practice. To our knowledge, this is the first study to compare the efficiency of no investigation versus ultrasound and versus MRI of the pelvis. However, given the sample size and retrospective design of the study, it is difficult to make clinical recommendation based solely on this study alone.

In addition, retrospective design and guideline-based criteria in selection of the cases in the assignment to a treatment group render a degree of selection bias. Although the small sample size may not allow drawing meaningful conclusions at this time, the number of cases of CEHA in our population appears to align with the reported incidence of the disease. The performance of MRI in management of endometrial hyperplasia would require additional randomised studies. However, MRI scan appears of value in the management of patients diagnosed with CEHA.

## Conclusions and significance of the study

In daily clinical practice, the management choices in patients’ evaluation of abnormalities of endometrial cavity pose significant diagnostic challenges for both radiologists and gynaecologists. As with this study, the role of MRI in evaluating suspected endometrial pathology remains uncertain and its usefulness is not yet clearly established. There is still an acute need of reliable, non-invasive methods to assist in lesion evaluation and establishing a diagnosis for appropriate triage of patients for more invasive diagnostic procedures and definitive management.

## Supplementary information

**Additional file 1: Table S1.** Histological features on endometrial hyperplasia with and without atypia (CEHA).

**Additional file 2: Figure S1.** Management and follow up histology of women from Group 1, who did not undergo any additional imaging studies.

## Data Availability

All data generated or analysed during this study are included in this published article and its supplementary information files.

## Abbreviations

CEHA: Complex endometrial hyperplasia with atypia
DMPA: Depot medroxyprogesterone acetate
EEC: Endometrial cancer
MDT: Multidisciplinary team
MRI: Magnetic resonance imaging
NPV: Negative predictive value
PPV: Positive predictive value
TVUS: Transvaginal ultrasound scan
US: Ultrasonography
