# Supplementary material for: The value of MRI in management of endometrial hyperplasia with atypia
Source: World J Surg Oncol. 2020 Feb 10;18:34. doi: 10.1186/s12957-020-1811-5 (PMC7011375; doi:10.1186/s12957-020-1811-5)
Supplement: Supplementary file 1 — Additional file 1: Table S1. Histological features on endometrial hyperplasia with and without atypia (CEHA). [file 12957_2020_1811_MOESM1_ESM.pdf]

Supplemental table 1. Histological features on endometrial hyperplasia with and without atypia (CEHA)

Microscopic features	Endometrial hyperplasia without atypia	Endometrial hyperplasia with atypia (CEHA)
Glandular Changes<br>(both will have increase gland to stroma ratio)	Mild to moderate increase in gland to stromal ratio.	Increase in gland to stromal ratio
Cytological changes	Cigar-shaped, oval, pseudo-stratified nuclei with smooth contours.<br>Uniform chromatin distribution.<br>Small to indistinct nucleoli.<br>Mitotic activity variable.<br>Amphophilic cytoplasm.	Rounding of nucleus, stratification with loss of polarity<br>Coarse chromatin<br>Prominent nucleoli.<br>Mitotic activity variable.<br>Eosinophilic cytoplasm.
Glandular confluence/ stromal exclusion/cribriform pattern<br><br>(Same finding in both; complexity of the architecture was used to differentiate between simple and complex hyperplasia)	Glands - irregular, variable size and some dilated. Branching, infolding and out pouching.<br><br>Simple hyperplasia–haphazardly spaced in abundant stroma.<br><br>Complex hyperplasia–closely packed with decreased stroma and highly irregular outlines	Glands - irregular, variable size and some dilated. Branching, infolding and out pouching.<br><br>Simple hyperplasia–haphazardly spaced in abundant stroma.<br><br>Complex hyperplasia–closely packed with decreased stroma and highly irregular outlines
