# Supplementary material for: The value of MRI in management of endometrial hyperplasia with atypia
Source: World J Surg Oncol. 2020 Feb 10;18:34. doi: 10.1186/s12957-020-1811-5 (PMC7011375; doi:10.1186/s12957-020-1811-5)
Supplement: Supplementary file 2 — Additional file 2: Figure S1. Management and follow up histology of women from Group 1, who did not undergo any additional imaging studies. [file 12957_2020_1811_MOESM2_ESM.pdf]

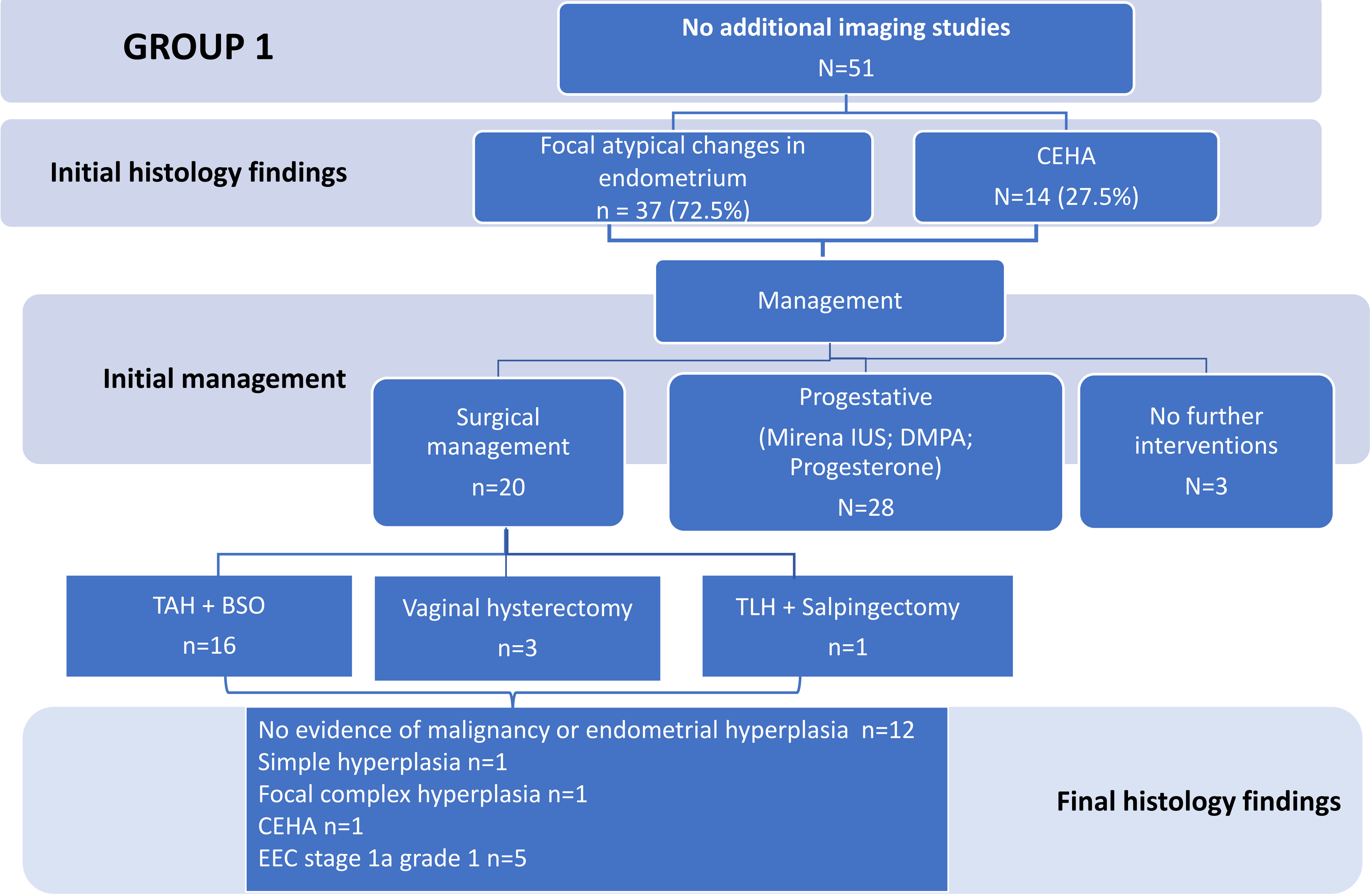
GROUP 1
No additional imaging studies
N=51
Initial histology findings
Focal atypical changes in endometrium
n = 37 (72.5%)
CEHA
N=14 (27.5%)
Management
Initial management
Surgical management
n=20
Progestative
(Mirena IUS; DMPA; Progesterone)
N=28
No further interventions
N=3
TAH + BSO
n=16
Vaginal hysterectomy
n=3
TLH + Salpingectomy
n=1
No evidence of malignancy or endometrial hyperplasia n=12
Simple hyperplasia n=1
Focal complex hyperplasia n=1
CEHA n=1
EEC stage 1a grade 1 n=5
Final histology findings
